# Antibiotic resistance: A cross-sectional study on knowledge, attitude, and practices among veterinarians of Haryana state in India

**DOI:** 10.14202/vetworld.2019.258-265

**Published:** 2019-02-14

**Authors:** Thulasiraman Parkunan, Manju Ashutosh, Bharathy Sukumar, Jatinder Singh Chera, Sendhil Ramadas, B. Chandrasekhar, S. Ashok Kumar, Rachana Sharma, M. Santhosh Kumar, Sachinandan De

**Affiliations:** 1 Animal Physiology Division, ICAR-National Dairy Research Institute, Karnal, Haryana, India; 2 National Institute of Epidemiology, Indian Council of Medical Research, Chennai, Tamil Nadu, India; 3 Animal Genomics Lab, Animal Biotechnology Centre, ICAR-National Dairy Research Institute, Karnal, Haryana, India; 4 Department of Agricultural Statistics, ICAR-Indian Institute of Wheat and Barley Research, Karnal, Haryana, India; 5 Dairy Microbiology Division, ICAR-National Dairy Research Institute, Karnal, Haryana, India; 6 Dairy Extension Division, ICAR-National Dairy Research Institute, Karnal, Haryana, India

**Keywords:** animal husbandry, antibiotic resistance, attitude, knowledge, practices, veterinarians

## Abstract

**Aim:**

The current study aimed to assess the knowledge, attitude, and practices pertaining to antibiotic usage among the field veterinarians who serve as nodal officers playing a crucial role in disseminating knowledge to the farmers regarding livestock management practices in India.

**Materials and Methods:**

A pilot study was conducted in which 106 of the 173 field veterinarians of Haryana, India, agreed to contribute through their valuable participation in the study. The collected data were critically analyzed by simple descriptive statistics, and the responses were ranked using Garrett’s ranking method.

**Results:**

Our study found that most of the clinicians were aware of the fundamental clinical aspects of antibiotic resistance (AR), i.e., the general causes and transmission of resistance, response during treatment failure, and safe disposal of hospital waste. Further, implementation of “antibiotic stewardship” (rational/responsible use of antibiotics) and interruption of AR transmission by means of cross-kingdom pathogens are two ways to restrict the spread of resistant pathogens which were not in the clinical purview of majority of the clinicians. This highlights a lack of awareness and scope of improving clinician’s knowledge pertaining to AR. Moreover, we got to know the methodology adopted by farmers for disposal of infected milk from diseased udders as well as their attitude toward diseased and unproductive animals.

**Conclusion:**

This study provides snippets of the current animal husbandry practices prevalent at the field level which would assist to plug in the gaps of knowledge regarding AR among the veterinarians as well as the general public and serve to reduce its deleterious impacts in Indian animal farming as well as in the world through the concept of “One World, One Health.”

## Introduction

Antibiotic resistance (AR) is a burning health-care issue influencing both humans and domestic animals across the globe. Antibiotics are widely used in animal husbandry sector for therapeutics and non-therapeutic purposes [1]. In food animal production, treating individual animal is practically impossible, rather rendering treatment to the entire livestock by medicating them with low dose of antimicrobials through feed, water, or parenteral routes is a better alternative. These mass medication procedures are collectively called metaphylaxis. In addition, prophylaxis is also another way to provide antibiotic treatment to animals [2].

To meet the mounting demands of animal protein for the growing population coupled with the increased purchasing power of developing nations, producers use antibiotics at subtherapeutic doses to compensate for unhygienic animal rearing practices followed during their production for short-term gains on growth. However, such faulty rearing practices have far-reaching implications such as the emergence of AR bacteria that limit the possibilities to cure human and animal illnesses through the administration of antibiotics, resulting into increasing economic losses for farmers as well as dairy processors and above all causing widespread environmental contamination [3,4]. Antibiotics commonly given to the animals are the same as those given to humans which are the foremost reason for depletion of their effectiveness, thereby jeopardizing human health and thus making AR a cause of global concern [5].

In India, irrational use of antibiotics, over-the-counter (OTC) sale of antibiotics, counterfeit drugs, incomplete control of infectious agents, and inadequate sanitary conditions all together increase the emergence and incidence of resistant and extensively resistant pathogens which transmit their resistance determinants between humans and animals through direct contact, food products, and environment [5-7]. Various control strategies such as awareness generation regarding AR in society have been initiated to reduce the deleterious effects of AR in India [6]. Awareness regarding proper scientific animal husbandry practices can act as a strong preventive measure to curtail AR.

Globally, India stood 4^th^ in antibiotic consumption (3%) for livestock production in 2010, which has led to an increase in AR at an alarming rate [3]. Thus, it was found very much necessary to assess the knowledge, attitude, and practices (KAP) on antibiotic use and resistance in animal husbandry (veterinarians) sector in India. Further, no attempts have been made in India to conduct studies regarding the usage of antibiotics in Veterinary medicine and also investigate the probable role of physicians and pharmacists in AR development and control.

The current study aimed to assess the knowledge, attitude, and practices pertaining to antibiotic usage among the field veterinarians who serve as nodal officers playing a crucial role in disseminating knowledge to the farmers regarding livestock management practices in India.

## Materials and Methods

### Ethical approval and Informed consents

There were no live animals used in this study, so there is no ethical approval necessary. Written consent was also obtained from the participants before administering the questionnaire.

### Background of respondents

Respondents selected for the survey were Government-appointed veterinarians from the state of Haryana, India. Haryana is located in the North region of India which is the highest milk consuming state in the country. A total of 173 veterinarians were approached for data collection, and of those, 106 (n) agreed to participate in the study that gave a response rate of 61.27%.

### Study tools and data collection

A questionnaire for this survey was developed by our research group based on prior literature review. It had 14 questions which were subdivided into six categories: Causes (2 items), transmission (2 items), diagnostics (1 item), treatment failure (2 items), waste disposal (2 items), and control strategies (5 items) related to AR with special reference to mastitis. The validity of the items in the questionnaire was assessed by a group of local experts and pre-tested with five randomly selected subject matter specialists who had similar characteristics with the study population. Details of the questions and their corresponding responses are provided in Tables-1 and 2.

**Table-1:** Details of questions with their respective options and the responses.

Categories	Q. No	Questions	Level	Number of responses

				Frequency (%)
Causes	Q1	Are you aware about the addition of antibiotics in the feed/water, provided to animals/birds?	Yes	86 (81)
			No	0 (0)
			Not answered	20 (19)
Transmission	Q3	Do you know how humans acquire resistant bacteria?	Direct contact with other humans and animals	27 (21)
			Animal product (milk, meat, egg)	25 (20)
			Contaminated water and soil	2 (2)
			Mobile genetic elements	6 (5)
			All the above	58 (46)
			Not answered	8 (6)
	Q4	Do humans acquire resistant genes/bacteria through plant products (fruits and vegetables)?	Yes	46 (43)
			No	55 (52)
			Not answered	5 (5)
Diagnostics	Q5	Are farmers ready to go for laboratory tests (culture sensitivity test)?	Yes	84 (79)
			No	16 (15)
			Not answered	6 (6)
Treatment failure	Q6	What is your next step when your treatment fails and the animal is not cured?	CST/Expert advice/Immunity	74 (70)
			Sell the animals	12 (11)
			Not answered	20 (19)
	Q7	What farmers will do to the animal when it is not responding to treatment?	CST/Alternate therapy/Change physician	32 (30)
			Sell the animals	58 (55)
			Not answered	16 (15)
Waste disposal	Q8	How are you disposing your hospital wastes?	Burial/incineration method	76 (72)
			Garbage/gutter	16 (15)
			Not answered	14 (13)
	Q9	How will you advise to dispose the mastitis milk?	Heat/Chemical treatment	12 (11)
			Sewage	52 (49)
			Not specified	14 (13)
			Not answered	28 (26)
Control strategies	Q10	Do you know what are the enhanced sewage and effluent treatment facility and phage therapy?	Yes	52 (49)
			No	42 (40)
			Not answered	12 (11)
	Q11	Do you think educating health professional and public will reduce the intensity of the problem?	Yes	98 (92)
			No	6 (6)
			Not answered	2 (2)
	Q12	Are you aware of the term “Antibiotic Stewardship?”	Yes	8 (8)
			No	96 (91)
			Not answered	2 (1)
	Q13	Do you know how to reduce the antibiotic usage in animal and human sector?	Yes	66 (62)
			No	34 (32)
			Not answered	6 (6)
	Q14	If yes for Q13, please provide the details	Judicious prescription	23 (28)
			Stop Quackery practices	17 (20)
			Extension-related activities	11 (13)
			Strict law implementation	9 (11)
			Immunity building and better livestock management	9 (11)
			Environmental health	5 (6)
			Selected antibiotic producers	1 (1)
			Alternative medicine	2 (2)
			Easy availability of laboratory	6 (7)

**Table-2:** Questions with the options and their corresponding ranks and GMS.

Categories	Q. No	Questions	Level	Number of responses	

				Rank	GMS*
Causes	Q2	According to you, what are the major causes of antibiotic resistance in India?	Irrational use	1	50.52
			Over the counter	2	23.44
			Waste disposal	3	11.24
			Counterfeit drugs	4	10.29

* GMS=Garrett’s mean score

The pre-tested questionnaire was self-administered to the respondents enrolled in this study from November 2016 to May 2017. The respondents were assured that their participation was voluntary and anonymous. The types of response alternatives were yes/no, multiple choice, ranking, and open-ended questions addressing the awareness cum knowledge of respondents on AR. However, some of the respondents skipped questions, and their corresponding responses were considered as a separate option in Table-1 as “not answered.”

### Statistical analysis

The data were analyzed by Crosstabs (simple descriptive statistics) using IBM SPSS version 20 software package. Results were expressed in frequencies and percentages. Garrett’s ranking method (most widely used technique) was used to rank the responses for Q2 (Table-2) [8].

Garrett’s formula for converting ranks into percentage is given as follows:

Percentage position = 100×(R_ij_−0.5)/N_j_

Where,

R_ij_ is the rank given for i^th^ factor by j^th^ respondent, and N_j_ is the number of factors ranked by j^th^ respondent. The percentage position of each rank is converted into scores by referring the Garrett’s and Woodworth’s table [9]. For each factor, the scores of individual respondents are added together and divided by the total number of the respondents for whom the scores are added. The mean scores for all the factors are then arranged in descending order and ranks are given to identify the important factors for policy implications.

## Results

### Causes of AR

Perusal of Table 1 response indicated that 81% of the respondents agreed that the farmers were aware about the addition of antibiotics in feed or water provided to animals/birds (Q1-Table-1). In Q2, the Garrett’s mean score (Table 2) for the irrational use of antibiotics was 50.52 and 23.44 for OTC use, 11.24 for improper waste disposal, and 10.29 for counterfeit drugs based on which ranking was carried out.

### Transmission of AR

From the survey, it was found that 21% of the respondents believed that the main mode of transmission of resistant bacteria to the humans was through the direct contact between humans and animals, while 20% considered the consumption of animal products as the major route. Some believed that the contaminated environment (2%) and mobile genetic material (5%) were also the factors that are responsible for the transmission. On the other hand, 46% of the respondents considered that all the above factors were contributing to the transmission of resistant bacteria to humans (Table-1-Q3). To check whether veterinarians were abreast with the recent happenings in the science of AR, we questioned their awareness about transmission of resistant bacteria/determinants through plant products (Q4), to which a majority of the respondents (52%) gave an affirmative reply, whereas the remaining 43% responded negatively (Table-1).

### Role of diagnostics in AR

Improper diagnosis of diseases is the major professional lacuna that is responsible for the misuse of antibiotics. In veterinary field, professional diagnosis mainly relies on laboratory tests. In this context, we asked the veterinarians if the farmers were willing to spend money on laboratory diagnosis, i.e., especially on culture sensitivity tests (CSTs) (Q5). Of the total responses, 79% of the respondents agreed that their farmers were in favor of laboratory diagnosis, while 15% disagreed with this (Table-1).

### Treatment failure

We asked the respondents about the plan of action in case of treatment failure (Table-1-Q6), and for this, the majority (70%) replied that they would seek expert opinion or opt for CST, whereas 11% would consider advising their farmers to sell the affected animals. The same question was asked to know the perception of the farmer (Q7) regarding treatment failure. 55% of the veterinarians who responded thought that their farmers would prefer selling the infected animals at the local market, while 30% thought that the farmers would opt for CST/alternate therapy/change veterinarian (Table-1).

### Waste disposal

To acquire information about waste disposal, we asked Q8 and Q9 regarding disposal of hospital wastes and milk collected from cows with mastitis, respectively (Table-1). For Q8, 72% of the respondents opined that burial/incineration was the best way to dispose hospital wastes, while 15% replied that they would prefer to dispose wastes directly through garbage/gutter. For Q9, 49% of the respondents considered advising their farmers to dispose the mastitis milk directly through sewage, whereas 11% would go for heat/chemical treatment of the milk before disposal through sewage. Around 13% gave non-specific answers by merely mentioning proper disposal without any specific details, and 26% did not answer.

### Control strategies

To test knowledge about different strategies to control AR, five questions (Q10-14) were put forth in the questionnaire (Table-1). Q10 asked regarding awareness about enhanced sewage and effluent treatment facilities as well as phage therapy. 49% of the respondents said that they were aware about these strategies, while 40% did not know about them and 11% did not answer. When asked about whether educating the health professionals and public would help to tackle AR in Q11, 92% of the respondents agreed, 6% disagreed, and 2% did not answer. In Q12, the respondents were checked for their awareness about the terminology “antibiotic stewardship.” As high as 91% of the respondents were unaware of this terminology, while 8% were aware about it, and 1% did not answer. In Q13, probable methods to reduce antibiotic usage in animal and human sector were asked to get practical ideas from the respondents regarding the reduction of antibiotic use. 62% of the respondents replied that they knew how to reduce antibiotic usage, and of these, 28% were in favor of judicious prescription of antibiotics, 20% for stoppage of quackery x 13% for extension-related activities, 11% suggested implementation of strict laws to monitor drug sale, while another 11% proposed immunity building and better livestock management. A few respondents recommended the maintenance of clean environment, alternative medicine, and easy availability of laboratories for diagnosis.

## Discussion

The Northwestern region is the highest milk consuming region in the Indian subcontinent. Due to limited budget and time constraints, our cross-sectional study was restricted in the Haryana state, whose milk consumption is high among the Northwestern states of India [10]. The response rate for the study was only 61.27% (106/173) which might be due to the busy schedule of the veterinarians or their lack of interest in participation. Based on the responses from the respondents, the following interpretations were made.

### Causes of AR

Although many countries have banned the irrational use of antibiotics as growth enhancer/feed supplement, the rising incomes and growing populations are the prime drivers for an increase in demand for animal products in India. In an intensive food/dairy animal farming system, producers often rely on antibiotics that serve as a stopgap in lieu of improving hygiene, husbandry conditions, and sanitation. There is sporadic use of antibiotics in large-scale production setups to promote growth and simultaneously prevent infection [5]. In the present study, the results showed that majority of the respondents were aware about the addition of antibiotics in feed/water (Q1-Table-1). These antibiotics are added at lower doses in different forms as a means to reduce harmful pathogens in the feed and to minimize the sequelae of latent infection in broiler/food animals [11].

Poor socioeconomic status, overcrowding of patients, inadequate prescription, overprescribing, and improper selection of antibiotics are the major reasons behind AR development among the public health sector [12]. Alternatively, literature reports that irrational usage of antibiotics, poor infection control in hospitals and community, inadequate sanitary conditions, OTC sale of antibiotic, and availability of counterfeit drugs in the market are the major causes of AR, which were also noted in the current study [13,14]. To check the current knowledge of respondents, we asked them to rank the major causes of AR in India (Q2-Table-2), and their responses were ranked as follows [9]. Absurd usage of antibiotics was ranked first, followed by OTC antibiotic availability (2^nd^ rank), waste disposal was adjudged as 3^rd^, and counterfeit drugs was given the 4^th^ preference, and these results are in accordance with the previous reports from India and Karachi [15,16].

There are hardly any government regulations promulgated to monitor antibiotic sale and use in India. There are procedural lapses in implementation of policies designed for the hospitals. Further, there are neither sufficient penalties for irrational prescription of an antibiotic nor sufficient manpower for regulating misuse and overuse of drugs [17]. These can lead to serious consequences in the present as well as in near future, as even bacterial strains causing general infections are becoming resistant to current antibiotics.

### Transmission of AR

The amount and pattern of antibiotics used in food animals are the major determinant for the propagation of resistant bacteria in the animal reservoir, and this positively correlates to the patterns and levels of drug usage. However, other determinants also play a crucial role in this transmission such as spread of resistant bacteria between animals that transfer AR to the common flora within the animal reservoir through mobile genetic elements [18].

Transmission of AR factors or the resistant bacteria from animals to humans can occur through various routes. In our study, around 20% of the responses (Q3 of the questionnaire-Table-1) were in favor of foodborne route, which is the most important/common route for the spread of resistant enteric bacterial pathogens. Similar responses were also recorded for direct contact between animals and humans. Bacteria, as well as antibiotic residues from food animal, spread widely in the environment including soil, through dung and urine which affect other bacteria in the environmental flora and fauna. Thus, these can become reservoirs of resistant bacteria and a source of their reintroduction into the food animal and human reservoirs [19], resulting in outbreak of infection. On the contrary, very few respondents thought of environmental contamination and mobile genetic material as the mode of AR transmission. However, many of them considered all the above routes as important for the transmission of antimicrobial resistance (AMR) corroborating the earlier findings [20].

To check whether the knowledge of veterinarians was up to date, we asked questions regarding AR transmission through plant products (Q4-Table-1). It was observed that 43% of the respondents were aware of such transmission, indicating that there has been growing knowledge of some veterinarians; however, most of them (52%) were still unaware about this route of transmission. The pathogens transmitted in such a manner are called cross-kingdom pathogens. In this cross-kingdom interaction, the physiology of both partners contributes to the outcome of the interaction (i.e., colonization of plants) [21,22]. Under certain conditions, resistant bacteria can survive on, penetrate into, and colonize internal plant tissues which may result in transmission of resistant bacteria to humans during their consumption [23].

### Role of diagnostics in AR

Disease diagnosis relies on a combination of farmer’s knowledge and availability of diagnostic tools. Availability and cost of tests for the animals’ side seem to limit their use, particularly, when the results of these tests cannot be extrapolated to the entire herd and may also need to be frequently repeated [24]. For individual animal treatment (internal medicine), additional costs of laboratory testing substantially increase the total costs of disease treatment which creates a burden on the farmers. The prime objective of the farmers is to reestablish the milk production in the shortest possible period rather than eliminate the pathogens [25,26]. In contrast, our study showed that around 79% of the respondents claimed that the farmers were ready to go for laboratory tests such as CST (Q5-Table-1). This indicates that the farmer’s attitude and behavior have been changed by giving importance to laboratory tests. In addition, there is an urgent need for the development of specific field diagnostic test kits for the detection of diseases which would help in choosing the correct antibiotic, promote their rational use, and also prevent infection.

### Treatment failure

Treatment failure occurs due to the emergence of resistant pathogens as a result of misuse (wrong antibiotic choice, inadequate source control, etc.) [27] and overuse of antibiotics; long-term low-level exposure is more detrimental than short term and full dose of antibiotics administered for therapeutic use [28].

Two open questions (Table-1) based on behavior/attitude were asked from treatment failure category, for which the respondents answered to Q6 and Q7 by considering themselves as both, a physician and a farmer. As a physician, majority of them would opt for expert opinion or CST, whereas a very few advised to cull/sell the animals. On the other hand, as a farmer, majority of them indicated that they would sell the animals in local market, while some of them would opt for CST/alternate therapy/change in physician options. The possible reasons for such an attitude among the farmers may be that the poor farmers are not able to shell out money to carry out expensive tests such as CST which needs to be performed before treatment to select the appropriate antibiotic, and hence, they find selling the infected animal in the local market as a better alternative. This leads to the spread of infection from one place to another. However, in India, majority of the farmers depend on livestock and farming for their daily needs, so expecting them to maintain unproductive animals does not seem logical, and hence, they try to sell such animals to avoid financial burden. Hence, looking into the grave situation, the government should make attempts to support the farmers by subsidizing the costs for laboratory diagnostics by providing ample funds [29].

### Waste disposal

According to the WHO, waste disposal should be conducted through thermal, chemical, biological, and mechanical processes as well as irradiation technologies [30]. In our study, majority of the respondents told (Q8-Table-1) that they would dispose hospital wastes by proper methods (72%) such as burial or burning/incineration. However, a few (15%) of the respondents said that they would use improper methods such as discarding the wastes directly in the garbage/gutter. This indicates that they may not be aware of the proper hospital waste disposal methods or it is possible that they are lacking the facility for doing so. A similar kind of study conducted in Orissa, India [31], reported that proper disposal procedures were not followed to dispose the hospital wastes and expired medicines from shops which led to pollution of water, grasslands, and air. Hence, it is important to enforce environmental laws or policies for the safe disposal of pharmaceutical wastes.

Milk from infected cows suffering from mastitis should neither be consumed nor be sold and must be withheld for the period recommended by the drug manufacturer. In this regard, we asked the veterinarians that what farmers did with the waste milk from infected/treated cows (Q9-Table-1), for which majority of them responded that the farmers would discard the milk directly into the sewage/gutter (49%), whereas 11% stated that the farmers would dispose it through heat/chemical treatment, while some of the respondents (26%) skipped the question. This showed that the farmers are unaware about the consequences of improper disposal of milk from infected/treated animals. Improper waste milk disposal may contaminate the environment (water and soil), resulting in the transmission of resistant determinants (mobile genetic elements/factors) from resistant bacteria to normal/commensal bacteria that are otherwise susceptible to various antibiotics [19,20,32]. Therefore, there are possibilities that they may also enter the food chain, and hence, to break the cycle, it is advised to boil the milk before discarding.

### Control strategies

The major factors responsible for AR are inappropriate prescription practices, inadequate patient education, limited diagnostic facilities, unauthorized sale of antibiotics, lack of appropriate functioning of drug regulatory mechanisms, and non-human use of antibiotics such as in animal production [14]. Before implementing the control strategies, these factors need to be taken into consideration because antibiotic use is inevitable in developing nations like India, due to various reasons such as poor sanitation, increased standard of living, and higher bacterial incidences [3,33,34].

To reduce the usage of antibiotics, various control measures have to be devised and implemented accordingly such as treatment before waste disposal, educating health professionals and public, alternative therapies such as phage therapy, implementing antibiotic stewardship, phasing out of subtherapeutic use of antibiotics in animal production, and gaining political and financial support from policymakers [13,14,35].

Veterinarians play a key role in implementing this above-mentioned preventive strategy, and hence, we asked a few questions related to control strategies in this study. In Q10 (Table-1), it was seen that many of the respondents were well aware (49%) of waste disposal treatment facility, but almost equal number of respondents were unaware (40%) about it, whereas for Q11 (Table-1), a majority of them answered “Yes” (92%) which signifies the importance of awareness in the AR tackling drive. Indian government implemented a new scheme to emphasize clean environment named, Swachh Bharat Abhiyan (Clean India Mission), which focus on better sanitation and hygiene as well as to create awareness about the prevention of infections among the public to reduce antibiotic usage [36]. Antimicrobial stewardship, a term coined by McGowan and Gerding in 1996 at Atlanta which gained significance after a decade in 2007 [37], is a coordinated program that promotes the systematic use of antibiotics that improve the patient’s outcomes by reducing the microbial resistance and the spread of infections caused by multidrug-resistant organisms. Many of the respondents (91%) were unaware about the term “Antibiotic Stewardship” (asked in Q12-Table-1) which indirectly showed that they failed to update their knowledge on current control strategies for AR. India also devised its own National Action Plan on AMR in 2017 which gives 4^th^ priority to antibiotic stewardship [38].

Finally, we asked questions (Q13 and 14-Table-1) related to the reduction of antibiotic usage in animal and human sector. Among the responses, the top two responses for reducing antimicrobial usage were judicious prescription (28%) and check on quackery (20%) which can be implemented by developing low-cost rapid diagnostics methods, promoting antibiotic stewardship, strict implementation of existing laws on quackery practitioners and selling of antibiotics on prescription. Further, 13% of the respondents believed that extension-related activities would have a profound impact in generating awareness through Information, Education, and Communication and by providing training to all the stakeholders (veterinarians, public, politicians, etc.). The extension-related activities have been given the first priority in India’s National Action Plan as well as in the WHO’s Global Action plan on AMR [6,38].

Hence, for the promulgation of “One World, One Health” concept, a strong collaboration between humans, animals, and environmental experts needs to be established [39,40].

## Conclusion

Veterinarians play a key role in safeguarding both human and animal health by improving animal husbandry practices, in particular, and public health, in general. However, scanty reports are available on awareness studies related to KAP on AR in animal husbandry (veterinarians). Hence, the current study was an attempt to plug in the gaps and to cover up the paucity of data pertaining to awareness study on KAP. Our snapshot study assessed the current awareness cum knowledge level of field veterinarians in the state of Haryana (India). It was considered essential to test the awareness of veterinarians through such kind of awareness studies as they are the nodal officers responsible for disseminating knowledge to the farmers. Further, it is of utmost importance to educate and train these veterinarians on priority basis to tackle AR to enhance the quality of health of the animals as well as the consumers. We are of the opinion that it is high time that such type of the study has to be replicated and conducted extensively all over the country to know the exact scenario of AR.

## Author’s Contributions

TP, MA, and SD contributed in the conception and design of the study. TP, BC, SAK, and RS contributed in the acquisition of the data. TP and SR contributed in the analysis and interpretation of data. BS, TP, JSC, and MSK contributed in drafting the article and revising it critically for important intellectual content. All authors read and approved the final manuscript.
